# HyperAttention and Linformer-Based β-catenin Sequence Prediction For Bone Formation

**DOI:** 10.7759/cureus.68849

**Published:** 2024-09-07

**Authors:** Pradeep Kumar Yadalam, Ramya Ramadoss, Raghavendra Vamsi Anegundi

**Affiliations:** 1 Periodontics, Saveetha Dental College, Saveetha Institue of Medical and Technical Sciences (SIMATS) Deemed University, Chennai, IND; 2 Oral Pathology and Oral Biology, Saveetha Dental College, Saveetha Institue of Medical and Technical Sciences (SIMATS) Deemed University, Chennai, IND

**Keywords:** attention networks, bone formation, peptides, periodontal therapy, regeneration

## Abstract

Introduction

Beta (β)-catenin, a pivotal protein in bone development and homeostasis, is implicated in various bone disorders. Peptide-based therapeutics offer a promising approach due to their specificity and potential for reduced side effects. Attention networks are widely used for peptide sequence prediction, specifically sequence-to-sequence models. Hence, the current study aims to develop a HyperAttention and informatics-based β-catenin sequence prediction for bone formation.

Methods

β-catenin protein sequences were downloaded and quality-checked using UniProt and FASTA sequences using DeepBio (Deep Bio Inc., Seoul, South Korea) for predictive analysis. Data was analyzed for duplicates, outliers, and missing values. The data was then split into training and testing sets, with 80% of the data used for training and 20% for testing, and peptide sequences were encoded and subjected to algorithms.

Results

The HyperAttention and Linformer models perform well in predictive sequence, with HyperAttention correctly predicting 87% of instances and Linformer predicting 89%. Both models have higher sensitivity and specificity, with Linformer showing better identification of 91% of negative instances and slightly better sensitivity.

Conclusion

The HyperAttention and Linformer models effectively predict peptide sequences with high specificity and sensitivity. Further optimization and development are needed for optimal application and balance between positive and negative instances.

## Introduction

Beta (β)-catenin is a crucial protein in cellular processes, including embryonic development, cell adhesion, and cell signaling which also regulates gene expression through interaction with transcription factors of the T-cell/lymphoid-enhancer factor (TCF/LEF) family [1]. β-catenin's structure consists of three major domains: the N-terminal domain, the armadillo repeat domain, and the C-terminal domain. Its most well-studied function is its involvement in the canonical Wnt signaling pathway, stabilizing β-catenin and translating it into the nucleus. Dysregulation of this pathway has been associated with various diseases, including cancer. β-catenin interacts with other signaling pathways and regulatory proteins, such as actin cytoskeleton, p120-catenin, and the PAR polarity complex [2]. It can also interact with other transcription factors, co-activators, and co-repressors, influencing gene expression context-dependently. Further research into β-catenin's mechanisms and functions could provide therapeutic targets for various diseases, particularly cancer. The *Wnt *gene, originating from integrase-1 in mouse breast cancer and the wingless gene in Drosophila, is a key component of Wnt signaling pathways, regulating cell proliferation, migration, and tissue renewal in mammals, including extracellular signals and gene expression [3].

β-catenin is key in bone formation and skeletal development, regulating osteoblast differentiation and osteoclast activity. It is involved in the Wnt signaling pathway, which activates osteoblast differentiation and bone formation. Loss of β-catenin disrupts osteoblast differentiation and impairs bone formation. It also interacts with other signaling molecules and transcription factors involved in bone formation, such as the bone morphogenetic protein (BMP) pathway and receptor activator of nuclear factor kappa-Β ligand (RANKL) [4]. β-catenin also regulates osteoclastogenesis, the process of osteoclasts forming from precursor cells. Dysregulation of β-catenin signaling can lead to bone disorders like osteoporosis, osteosarcoma, and skeletal abnormalities. Targeting β-catenin and its associated signaling pathways could be a potential therapeutic approach for bone-related diseases.

Peptide sequence prediction is crucial for drug design due to their potential as therapeutics. Peptides are short amino acids that can interact with target proteins, making them attractive candidates for developing drugs that modulate protein function. Attention networks are widely used for peptide sequence prediction, specifically sequence-to-sequence models. These models can learn the relationships between the input protein sequence and the corresponding peptide sequence, generating new peptide sequences with desired properties. Peptide sequence prediction is crucial for discovering new peptides, optimizing properties, restructuring existing ones, and predicting peptide-protein interactions. Attention networks can generate new peptide sequences with specific binding affinity, optimize properties, and provide insights into peptide-protein interactions [5]. A previous study showed that the Epitope region prediction-based deep learning method with an attention mechanism improves epitope region prediction accuracy by considering the whole antigen protein's characteristics and the target sequence. A study developed a deep learning model called NeuroPred-CLQ, which outperformed state-of-the-art predictors in identifying neuropeptides with 93.6% accuracy and 98.8% area under the curve (AUC) on an independent test set [6].

Transformer-based Peptide-specific Lightweight Multi-Scale Graph Neural Network (TP-LMMSG) is a new graph deep learning model that enhances peptide chain structure investigation and amino acid information. It outperforms other antimicrobial peptide (AMP), antiviral peptide (AVP), and anticancer peptide (ACP) prediction models and addresses time-consuming pre-processing challenges in graph neural network frameworks [7]. Analyzing attention weights helps identify key regions or amino acid residues in protein sequences that interact with specific peptides, guiding design for improved binding affinity or selectivity. Transformer architecture, a natural language processing development, has influenced deep learning architectures and is applied to predict peptides. Peptide sequence encoding is separate from the protein encoding module, assuming it contains all the necessary information for binding. However, the peptide sequence alone may not determine bound conformation, as the same peptide can take on different shapes. Few studies focus on predicting peptide sequences for drug design and disease understanding. 

The HyperAttention and Linformer architectures are effective tools for peptide sequence prediction, accurately predicting protein-protein interactions, post-translational modifications, and structural characteristics related to bone formation. They use attention mechanisms to understand amino acid dependencies and handle long input sequences, capturing intricate patterns. Glycogen synthase kinase-3beta (GSK-3β) phosphorylates β-catenin without Wnt ligand, causing degradation and signaling inactivation. Wnt binding to coreceptors inactivates GSK-3β, stabilizes β-catenin, and affects gene expression in the nucleus. Dkk proteins bind to LRP5/6 and Krm, causing membrane depletion and inhibiting Wnt signaling. Sclerostin also inhibits Wnt signaling through binding to LRP5/6 independently [3,7]. This study used HyperAttention and Linformer models to analyze and predict the functional sequences of beta-catenin proteins, and structural characteristics, using deep learning and computational biology to understand bone formation.

## Materials and methods

This study utilized computational modeling techniques to analyze data. Ethical review was not required as the research involved no human or animal subjects.

Peptide sequence retrieval

UniProt is a database that provides extensive information on protein sequences and their annotations, integrating data from various sources. It helps researchers search for specific protein sequences, access functional information, and learn about genetic variations or cellular localization. UniProtKB includes reviewed protein sets, unreviewed sets, and cluster sequences based on sequence identity. It links to other databases like Protein Data Bank, PubMed, and Gene Ontology for enhanced protein data analysis.

Using UniProt [8], β-catenin protein sequences were downloaded using ID P26232, P35222, and Q9UI47. These sequences were identified and checked for quality. FASTA sequences were subjected to the DeepBio tool (Deep Bio Inc., Seoul, South Korea), HyperAttention, and Linformer algorithms for predictive analysis.

DeepBio

DeepBio [9] is a web service that allows researchers to develop deep-learning architectures for biological sequence data. It also supports nine base-level functional annotation tasks with interpretations and graphical visualizations. DeepBio allows fast prediction with large-scale sequence data and demonstrates accurate, robust, and interpretable biological sequence functional analysis predictions. After obtaining data, duplicates were removed, outliers and missing values were removed, and the data was then split into training and testing sets, with 80% of the data used for training and 20% for testing. Peptide sequences were into one hot encoding and subjected to algorithms.

HyperAttention architecture

Attention architecture is a deep learning model that incorporates attention mechanisms to enhance the learning and prediction capabilities of the network. The HyperAttention architecture comprises multiple attention modules with specific hyperparameters such as the number of attention heads, hidden size, and dropout rate. These parameters, learning rate, batch size, and training epochs, significantly improve the architecture's performance. The number of attention heads allows the network to capture a wider range of dependencies and increase computational complexity. The hidden size controls the dimensionality of output representations, determining the capacity and expressiveness of the model. The regularization technique calculated the dropout rate to prevent overfitting during training. Standard hyperparameters for training deep learning models were applied to the HyperAttention architecture, such as learning rate, batch size, and number of training epochs. These hyperparameters were tuned and optimized through techniques like grid search. The combination of hyperparameters depends on the specific task, dataset, and available computational resources used for predictive analysis. [10-12]

Hyperparameters of the Hyper Attention architecture include: (i) Number of attention heads: The parameter 'attention head' influences the network's diversity of dependencies, enabling a wider range of relationships but increasing computational complexity; (ii) Hidden size: The hyperparameter regulates the dimensionality of output representations produced by attention modules, thereby determining the model's capacity and expressiveness; and (iii) Dropout rate: The dropout rate is a regularization technique that reduces the model's reliance on specific features and improves generalization by randomly setting a fraction of attention layer outputs to 0.

The training of deep learning models involves considering other hyperparameters like learning rate, batch size, and number of training epochs. The learning rate controls the step size during gradient descent optimization, batch size determines the number of samples processed, and the number of training epochs indicates the number of iterations. Hyperparameters are optimized during model development to find the best combination for a task, using techniques like grid search to explore predefined values and computational resources.

Linformer architecture

The Linformer architecture is a variant of the Transformer model designed for processing long input sequences. The architecture also introduces several hyperparameters, such as hidden size, number of attention heads, input sequence length, and feed-forward dimension, which control the behavior and performance of the model. The hidden size hyperparameter determines the dimensionality of intermediate representations, while the number of attention heads determines the diversity and complexity of learned representations. The maximum sequence length hyperparameter sets an upper limit on the length of input sequences the model can process effectively. The feed-forward dimension hyperparameter controls the hidden layer's size in the feed-forward network [13,14].

The hyperparameters in the Linformer architecture include: (i) Hidden size: The hyperparameter in the Linformer architecture determines the dimensionality of intermediate representations, influencing the model's capacity and expressiveness; (ii) Number of attention heads: The hyperparameter, similar to the Hyper Attention architecture, influences the complexity and diversity of learned representations, enabling the model to capture intricate patterns and relationships; (iii) Input sequence length: The hyperparameter limits the Linformer model's processing capacity by setting an upper limit on the length of input sequences, preventing overburdening; and (iv) Feed-forward dimension: The hyperparameter in the Linformer architecture affects the size of the hidden layer in the feed-forward network component, thereby affecting the model's representational capacity and flexibility.

The Linformer architecture, optimized using grid search, was combined with DeepBIO and HyperAttention to form a comprehensive framework for functional annotation prediction.

## Results

**Figure 1:**
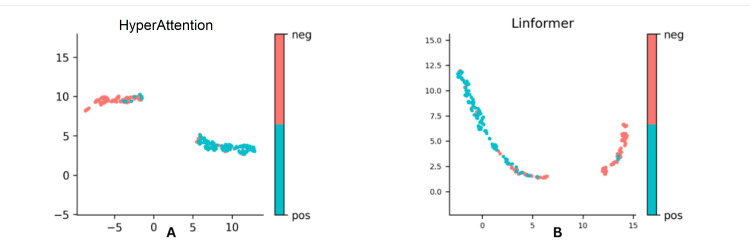
Distribution of attention scores for HyperAttention (A) and Linformer (B) models. Each panel displays the distribution of attention scores for a given model architecture. The x-axis represents the attention score, with negative values indicating negative attention and positive values indicating positive attention. The y-axis represents the frequency of attention scores within a given range. Red points represent negative attention scores, while blue points represent positive attention scores.

The HyperAttention and Linformer models show good performance on predictive sequence. The HyperAttention model correctly predicts 87% of instances, while the Linformer model has an accuracy of 0.89, indicating correct predictions for 89%. The Linformer model has a higher sensitivity of 0.87, indicating better detection of positive instances. The HyperAttention model has a specificity of 0.96, indicating better classification of 96% of negative instances, while the Linformer model has a specificity of 0.91, indicating better identification of 91% of negative instances. The AUC indicates better performance in distinguishing between positive and negative instances. The Linformer model has slightly better sensitivity but slightly worse specificity and AUC compared to the HyperAttention model, as shown in Table 1, Figure 1. 

**Table 1:** HyperAttention and Linformer performance metrics based on accuracy, sensitivity, specificity, and AUC Accuracy represents the overall correct prediction rate. Sensitivity (true positive rate) indicates the proportion of actual positive cases correctly identified. Specificity (true negative rate) represents the proportion of actual negative cases correctly identified. AUC is the area under the receiver operating characteristic curve, measuring the model's ability to discriminate between positive and negative classes.

Model Name	Accuracy	Sensitivity	Specificity	AUC
HyperAttention	0.87	0.78	0.96	0.928
Linformer	0.89	0.87	0.91	0.919

The performance evaluation curves used in machine learning to assess binary classification models: the receiver operating characteristic (ROC) curve and the Precision-Recall curve. The ROC curve plots the true positive rate (TPR) against the false positive rate (FPR) at different threshold settings, with higher AUC values indicating better model performance. The Precision-Recall curve plots precision against recall, with higher values indicating better performance. These curves are crucial for evaluating classification models, especially in situations with imbalanced classes or different false positives and negative costs. The performance across all classification thresholds helps in comparing different models.

Uniform Manifold Approximation and Projection (UMAP) shows data clustering and a technique that reduces high-dimensional data's global structure and neighborhood relationships in a lower-dimensional space. It uses metrics like trustworthiness, continuity, effective number of neighbors, and cross-entropy to evaluate the quality of dimensionality reduction, ensuring it accurately captures and reflects the original data's structure and relationships. It helps understand how well the reduced-dimensional representation captures and reflects the inherent structure and relationships in the original high-dimensional data.

Test accuracy and loss curves were plotted for both the HyperAttention and Liformer models across all epochs to assess their learning progress on the training data. These curves visualize the model's performance during training, comprising two plots: the Test Accuracy Curve and the Test Loss Curve. The Test Accuracy Curve illustrates the model's accuracy on the test dataset over each complete pass through the entire training dataset while the Test Loss Curve depicts how the model's error decreases with each training iteration up to 50 complete passes through the entire training dataset.

## Discussion

The Wnt/β-catenin signaling pathway comprises frizzled (FZD) proteins, low-density lipoprotein receptor-related protein 5/6 (LRP5/6), dishevelled (DvL), Axin (AXIN), adenomatous polyposis coli (APC), glycogen synthase kinase-3 beta (GSK-3β), casein kinase 1 alpha (CK-1α), β-catenin, and TCF/LEF. FZD proteins interact with Wnt ligands, LRP5/6 [4]. When the pathway is off, a β-catenin destruction complex forms. Twa1 promotes Wnt/β-catenin signaling by retaining β-catenin nuclear. FOXKs transport DVL into the nucleus, while ICAT, initially an inhibitor, blocks APC-mediated β-catenin degradation. These factors contribute to Wnt signal transduction and nuclear retention in the nucleus [15]. These factors influence the Wnt-β-catenin pathway for bone formation. The β-catenin sequence gene encodes a protein involved in adherens junctions (AJs), crucial for epithelial cell layers. It regulates cell growth, adhesion, and signaling cell division. Mutations in the APC gene are associated with colon, colorectal, and ovarian cancers. Alternative splicing produces multiple transcript variants, highlighting the importance of these genes in cancer prevention. Predicting sequences will solve complex biological problems and may give possible insights into bone formation [16].

The canonical Wnt signaling pathway, also known as the Wnt/β-catenin pathway, is crucial for cellular development, proliferation, and differentiation. It activates a signaling cascade when Wnt ligands bind to Fzd proteins, leading to the stabilization and nuclear accumulation of β-catenin. Without Wnt ligands, β-catenin is targeted for degradation by a destruction complex. However, binding to Fzd receptors disrupts this, allowing β-catenin to accumulate and translocate into the nucleus. The non-canonical Wnt signaling pathway, the Wnt/Ca pathway, modulates intracellular calcium levels and activates downstream effectors like protein kinase C and calcium/calmodulin-dependent protein kinase II. Fzd proteins, which are conserved across different species, interact with Wnt ligands and transmit downstream signals. Co-receptors, such as LRP5/6, also play essential roles in Wnt signaling [4].

Peptide sequence prediction [17,18] using attention networks is a sophisticated bioinformatics and computational biology method. These models use attention mechanisms to focus on crucial parts of the input sequence, capturing long-range dependencies and extracting meaningful features [19,20]. They can handle variable-length input sequences and provide interpretability by highlighting regions of the input sequence that influence predictions. However, training a model requires a large dataset of labeled peptide sequences and careful optimization and tuning. As bioinformatics advances, attention networks will likely play a significant role in unraveling complex relationships between peptide sequences and their functional properties. In a previous study, PepNN, a model that combines graph neural networks and transfer learning, was used to predict peptide-protein interactions by considering the flexibility and shape changes of peptides. It demonstrated strong performance on benchmark datasets and can make predictions without specific peptide information, facilitating the identification of new binding proteins. Following a similar approach, we employed hyperattention networks to predict peptide sequences [21].

Another study showed that the iAMP-Attenpred predictor is a novel AMP predictor that utilizes the BERT (Bidirectional Encoder Representations from Transformers) model for feature encoding in natural language processing and combines multiple models to discover AMPs. BERT is a powerful language model originally designed for natural language processing tasks. It is used to encode peptide sequences as if they were sentences. Each amino acid is treated as a "word," and BERT is trained to understand the relationships between these amino acids. The study uses AMPs and non-AMP sequences as words in a BERT pre-training model, combined with a composite model, iAMP-Attenpred, proving its effectiveness in AMP prediction similar to our Linformer-based prediction with an accuracy of 89%. A machine learning model predicts T-cell receptor binding to a specific peptide using amino acid sequences [22,23], showing competitive performance on benchmarks and external datasets. It associates neural network weights with protein structural properties, aiding in molecular recognition, which corroborates with our study design in predicting beta-catenin sequences with good accuracy,

The HyperAttention and Linformer models effectively predict peptide sequences but have different strengths and weaknesses. The HyperAttention model has high specificity, classifying 96% of negative instances, while the Linformer model has higher sensitivity, detecting 87% of positive instances. The Linformer model has slightly better overall accuracy, with 89% accuracy, but the HyperAttention model has a slightly higher AUC score. Future research should focus on improving the performance of these models, exploring architectural variations, hyperparameter optimization, and training strategies. However, attention-based models have limitations, such as reliance on large labeled datasets and interpretability of attention weights.

UniProt served as the primary repository for β-catenin protein sequences, which were subsequently subjected to rigorous quality control measures. The obtained FASTA sequences were then processed through DeepBio, HyperAttention, and Linformer algorithms to predict peptide sequences. DeepBio, a versatile platform for deep learning applications in biological sequences, facilitated model development and interpretation [9]. The HyperAttention architecture, incorporating attention mechanisms, was employed to capture intricate sequence dependencies, while the Linformer architecture was designed to efficiently handle long input sequences. Hyperparameter optimization, including the adjustment of attention heads, hidden sizes, dropout rates, learning rates, batch sizes, and training epochs, was crucial in enhancing model performance. However, while this study provides valuable insights into peptide sequence prediction using HyperAttention and Linformer models, it's essential to acknowledge certain limitations.

Firstly, the reliance on the UniProt database for protein sequence retrieval might introduce biases due to potential incompleteness or errors in the database [24]. A more comprehensive approach involving multiple databases could enhance data diversity and reliability. Secondly, the study primarily focuses on β-catenin sequences. While this protein is crucial, generalizing the findings to other protein families would require further investigation. Thirdly, the hyperparameters used for the HyperAttention and Linformer models were optimized based on standard practices. A more exhaustive hyperparameter tuning process could potentially lead to improved model performance. Lastly, the evaluation metrics employed in this study primarily focused on accuracy. Incorporating additional metrics like precision, recall, and F1-score would provide a more comprehensive assessment of model performance. Addressing these limitations in future research can contribute to the development of more robust and accurate peptide sequence prediction models.

Future research on HyperAttention and Linformer models can improve their performance by exploring architectural variations, hyperparameter optimization, and training strategies. Nevertheless, attention-based models face challenges such as reliance on extensive labeled datasets and the interpretability of attention weights. To mitigate these limitations, researchers should prioritize developing methods to address data scarcity, including semi-supervised or unsupervised learning approaches, and refining techniques to visualize and interpret attention weights. By overcoming these obstacles, the effectiveness of attention-based models in peptide sequence prediction can be significantly enhanced.

## Conclusions

β-catenin sequence prediction will help identify novel drugs for bone formation. The HyperAttention and Linformer models perform excellently in predicting peptide sequences, with the former demonstrating high specificity and the latter exhibiting higher sensitivity. The choice between these models depends on the application and desired balance between positive and negative instances. Further optimization and development of these attention-based models, including architectural variations, hyperparameter optimization, and computational techniques, are needed.
